# The complete mitochondrial genome of the cryptic species (Form II) in kuruma shrimp *Marsupenaeus japonicus* (Decapoda: Penaeidae)

**DOI:** 10.1080/23802359.2018.1437835

**Published:** 2018-02-10

**Authors:** Shengping Zhong, Yanfei Zhao, Xianfeng Wang, Zhifei Song, Qin Zhang, Xiuli Chen

**Affiliations:** a Key Laboratory of Marine Biotechnology, Guangxi Institute of Oceanology, Beihai, China;; b College of Ocean and Earth Sciences, Xiamen University, Xiamen, China;; c Guangxi Key Laboratory of Aquatic Genetic Breeding and Healthy Aquaculture, Guangxi Academy of Fishery Sciences, Nanning, China

**Keywords:** Mitochondrial genome, *Marsupenaeus japonicus*, Decapoda

## Abstract

The Kuruma shrimp *M. japonicus* is economic important species in the shrimp fishery and aquaculture. However, it is a species complex consisting of two cryptic species (Form I and Form II), which can be difficult to identify based on morphology. Here, we report the complete mitochondrial genome sequence of *M. japonicus* (Form II) from Beibu Bay. The mitogenome has 15,966 base pairs and made up of total of 37 genes (13 protein-coding, 22 transfer RNAs and 2 ribosomal RNAs), and a putative control region. There were 1029 mutations sites between these two cryptic species, and the most variable sites were putative control region. This study adds a distinct mitogenome of *M. japonicus* (Form II) and will provide useful genetic information for future genetic variation identification and genetic diversity evaluation of this economic valuable shrimp.

Kuruma shrimp (*M. japonicus*) (Decapoda, Penaeidae) is a commercially important crustacean species in Indo-West Pacific. Recent research has revealed the occurrence of cryptic species in the kuruma shrimp (Forms I and II), which shared very similar morphological traits and misidentifications have been occasionally reported (Tsoi et al. 2014). In recent years, the wild stocks of *M. japonicus*, however, have been declining steadily due to overexploitation and deterioration of coastal habitats that support juveniles (Hamasaki and Kitada 2006). The stock enhancement programs aim to recovery wild populations have been conducting in Japan since 1964. Accurate species identification and genetic structure assessment of *M. japonicus* are fundamental prerequisite for wild stock enhancement (Kalinowski 2010). In this study, we report the complete mitochondrial genome sequence of *M. japonicus* (Form II) from Beibu Bay, which will be an important genetic resource to assist in molecular identification of the phylogenetic position between the cryptic species in kuruma shrimp and genetic variation within *M. japonicus*.

The tissue samples of *M. japonicus* from 3 individuals was collected from Beibu Bay, China (Beihai, 21.419002 N, 109.256545 E), and the whole body specimens (#GQ0053) were deposited at Marine biological Herbarium, Guangxi Institute of Oceanology, Beihai, China. The total genomic DNA was extracted from the muscle of the specimens using an SQ Tissue DNA Kit (OMEGA, Guangzhou, China) following the manufacturer’s protocol. DNA libraries (350bp insert) were constructed with the TruSeq NanoTM kit (Illumina, San Diego, CA) and were sequenced (2 × 150bp paired-end) using HiSeq platform at Novogene Company, China. Mitogenome assembly was performed by MITObim (Hahn et al. 2013). The complete mitogenome of kuruma shrimp (Form I) from Japan (GenBank accession number: NC_007010) was chosen as the initial reference sequence for MITObim assembly. Gene annotation was performed by MITOS (Bernt et al. 2013).

The complete mitogenome of *M. japonicus* from Form II was found to be 15,966 bp in length (GenBank accession number: MG772559), consisting of the usual set of 13 protein-coding, 22 tRNA and 2 rRNA genes, and a putative control region. The overall base composition of the mitogenome was estimated to be A 32.7%, T 33.7%, C 20.5% and G 13.1%, with a high A + T content of 66.4%, which is similar, but slightly different from Form I (Yamauchi et al. 2004). There were 1029 mutations sites between these two cryptic species, and the most variable sites were putative control region. This result indicated the high genetic divergence between these two populations, which further supporting the occurrence of cryptic species in the kuruma shrimp (Forms I and II) (Tsoi et al. 2014). The result of phylogenetic tree of 10 species (including other 9 species from family Penaeidae in NCBI) supported the close relationship among genus *Marsupenaeus*, *Parapenaeopsis* and *Metapenaeus* (Figure 1), as they shared the same branch node with the highest bootstrap value. COX3 and NAD5 terminated with an incomplete stop codon T, which is thought to be completed with the addition of 3′ adenine residues to the mRNA (Ojala et al. 1981). The complete mitochondrial genome sequence of *M. japonicus* from Beibu Bay, adds a distinct mitogenome of *M. japonicus* (Form II), which will contribute to further phylogenetic and comparative mitogenome studies of this economic valuable shrimp.

**Figure 1.**
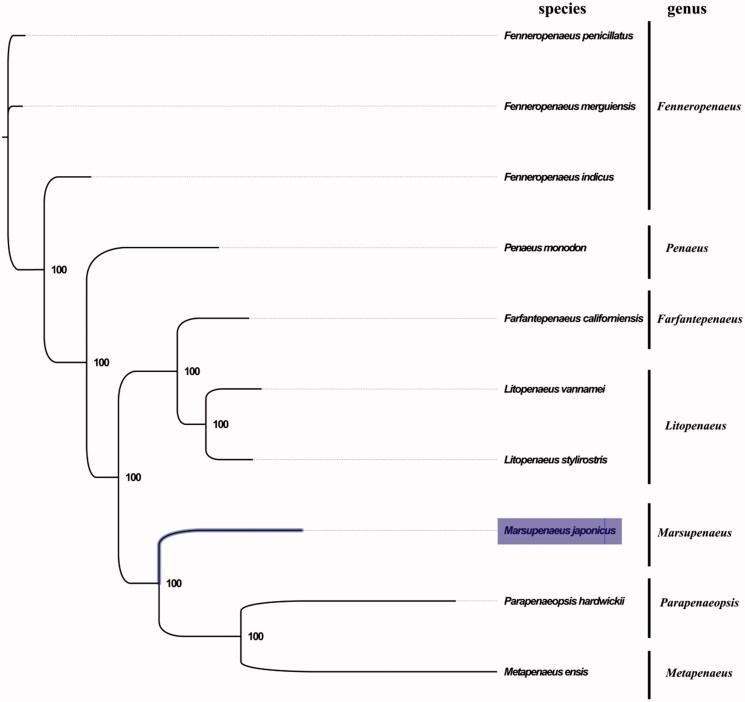
Phylogenetic tree of 10 species in family Penaeidae. The complete mitogenomes is downloaded from GenBank and the phylogenic tree is constructed by maximum-likelihood method with 100 bootstrap replicates. The bootstrap values were labeled at each branch nodes. The gene's accession number for tree construction is listed as follows: *Fenneropenaeus penicillatus* (NC_026885), *F. merguiensis* (NC_026884), *F. indicus* (NC_031366), *Penaeus monodon* (NC_002184), *Farfantepenaeus californiensis* (NC_012738), *Litopenaeus vannamei* (NC_009626), *L. stylirostris* (NC_012060), *Parapenaeopsis hardwickii* (NC_030277) and *Metapenaeus ensis* (NC_026834).
